# Changes in Metabolic Syndrome Status and Risk of Dementia

**DOI:** 10.3390/jcm9010122

**Published:** 2020-01-02

**Authors:** Ji Eun Lee, Dong Wook Shin, Kyungdo Han, Dahye Kim, Jung Eun Yoo, Jinkook Lee, SangYun Kim, Ki Young Son, Belong Cho, Moon Jong Kim

**Affiliations:** 1 Department of Family Medicine, CHA Bundang Medical Center, CHA University, Seongnam-si 13496, Korea; jieun10@gmail.com; 2 Department of Family Medicine, Samsung Medical Center, Sungkyunkwan University School of Medicine, Seoul 06351, Korea; 3 Department of Digital Health, SAIHST, Sungkyunkwan University, Seoul 06351, Korea; 4 Department of Biostatistics, The Catholic University of Korea, Seoul 06591, Korea; hkd917@naver.com (K.H.); dahaene1228@naver.com (D.K.); 5 Department of Family Medicine, Healthcare System Gangnam Center, Seoul National University Hospital, Seoul 06236, Korea; ujungeun@gmail.com; 6 Department of Economics & Center for Economic & Social Research, University of Southern California, Los Angeles, & RAND Corporation, Santa Monica, CA 90401-3208, USA; jinkookl@usc.edu; 7 Department of Neurology, Seoul National University Bundang Hospital & Seoul National University College of Medicine, Seongnam-si 13620, Korea; neuroksy@snu.ac.kr; 8 Department of Family Medicine, Asan Medical Center, Seoul 05505, Korea; mdsky75@gmail.com; 9 Department of Family Medicine, Seoul National University Hospital, Seoul 03080, Korea; belong@snu.ac.kr

**Keywords:** metabolic syndrome, dementia, hyperglycemia, blood pressure, prevention

## Abstract

This study investigated the effects of changes in metabolic syndrome (MS) status and each component on subsequent dementia occurrence. The study population was participants of a biennial National Health Screening Program in 2009–2010 and 2011–2012 in Korea. Participants were divided into four groups according to change in MS status during the two-year interval screening: sustained normal, worsened (normal to MS), improved (MS to normal), and sustained MS group. Risk of dementia among the groups was estimated from the second screening date to 31 December 2016 using a Cox proportional hazards model. A total of 4,106,590 participants were included. The mean follow-up was 4.9 years. Compared to the sustained normal group, adjusted hazard ratios (aHR) (95% confidence interval) were 1.11 (1.08–1.13) for total dementia, 1.08 (1.05–1.11) for Alzheimer’s disease, and 1.20 (1.13–1.28) for vascular dementia in the worsened group; 1.12 (1.10–1.15), 1.10 (1.07–1.13), and 1.19 (1.12–1.27) for the improved group; and 1.18 (1.16–1.20), 1.13 (1.11–1.15), and 1.38 (1.32–1.44) for the sustained MS group. Normalization of MS lowered the risk of all dementia types; total dementia (aHR 1.18 versus 1.12), Alzheimer’s disease (1.13 versus 1.10), and vascular dementia (1.38 versus 1.19). Among MS components, fasting glucose and blood pressure showed more impact. In conclusion, changes in MS status were associated with the risk of dementia. Strategies to improve MS, especially hyperglycemia and blood pressure, may help to prevent dementia.

## 1. Introduction

With populations aging worldwide, the number of people with dementia is steadily increasing. In 2015, the estimate of people with dementia was 47.47 million and the number is expected to reach 75.63 million in 2030 and 135.46 million in 2050 [1]. Dementia is a leading chronic disease that contributes to disability. Worldwide costs of dementia are high [2]. 

To date, considerable effort has been invested in preventing and curing dementia. However, no significant preventative or therapeutic drugs have been developed yet. Although acetylcholine esterase inhibitors and N-methyl-D-aspartate (NMDA) receptor antagonists have been used, they only slow the progression of dementia and cannot cure the disease. Therefore, at present, the most important way to manage dementia is to find and control risk factors to prevent the disease.

In this regard, some studies have shown that metabolic syndrome (MS) increases the risk of dementia and cognitive decline [3,4]. MS refers to a cluster of risk factors for cardiovascular disease that occur together more often than by chance alone [5]. The factors include hyperglycemia, raised blood pressure, elevated triglyceride levels, low high-density lipoprotein cholesterol (HDL) levels, and central obesity. 

However, the association between MS and dementia is not yet conclusive. Some studies have not found significant associations between MS and dementia [6,7]. In addition, in some studies, MS showed a protective effect for cognitive decline, especially in older adults [7,8]. A recent meta-analysis showed no statistically significant association between MS and Alzheimer’s disease (AD) or total dementia. MS increased only the incidence of vascular dementia (VD) [9]. 

Among the components of MS, hyperglycemia is most consistently associated with poor cognitive abilities [10,11,12]. The effects of other MS components are more heterogeneous. Blood pressure was negatively associated with Mini-Mental State Examination (MMSE) score in a study [10], however no significant impact on cognitive status was seen in another [11]. Central obesity was a risk factor for cognitive decline [8]. In another study, the association was not significant [11].

Furthermore, most studies to date examined MS status only once, at baseline. Only two studies examined the relationship between the results of repeated measures of MS status and incidence of dementia. A cohort study in Taiwan showed worsened MS associated with a higher risk of dementia [13]. However, the study did not examine the effect of each MS component. It also did not analyze the results according to dementia type. The sample size was relatively small (*n* = 3458) and dementia was defined only by International Classification of Diseases (ICD) codes, which may be entered incorrectly. Another study assessed MS three times over a 10-year follow-up period [4]. However, this study categorized MS status into only never, nonpersistent, and persistent. Therefore, the effects of improvement and deterioration were not observed.

Identifying if MS improvement is actually effective in preventing dementia and the components that affect it is important. Therefore, this study aimed to determine the effects of changes in MS and each component on subsequent dementia occurrence. We used data from repeated health screenings from a large, nationally-representative sample.

## 2. Materials and Methods

### 2.1. Data Source 

The Korean National Health Insurance (KNHI) service is a mandatory public health insurance system that provides universal health coverage to all Koreans. The KNHI service offers a biennial National Health Screening Program (NHSP) to the Korean population aged 40 and above, and all employees regardless of age. The screening program includes a questionnaire (past medical history, health behavior), anthropometric exam (body mass index, blood pressure), and laboratory tests (for example, blood sugar, cholesterol) [14]. 

The KNHI provides a National Health Information Database (NHID) comprising a complete set of the health information of 50 million Koreans, a medical treatment database (based on bills claimed by service providers for their medical expense claims), and a health examination database (NHSP results) and medical care institution database [15]. This study used the NHID. 

### 2.2. Study Population 

**Figure 1:**
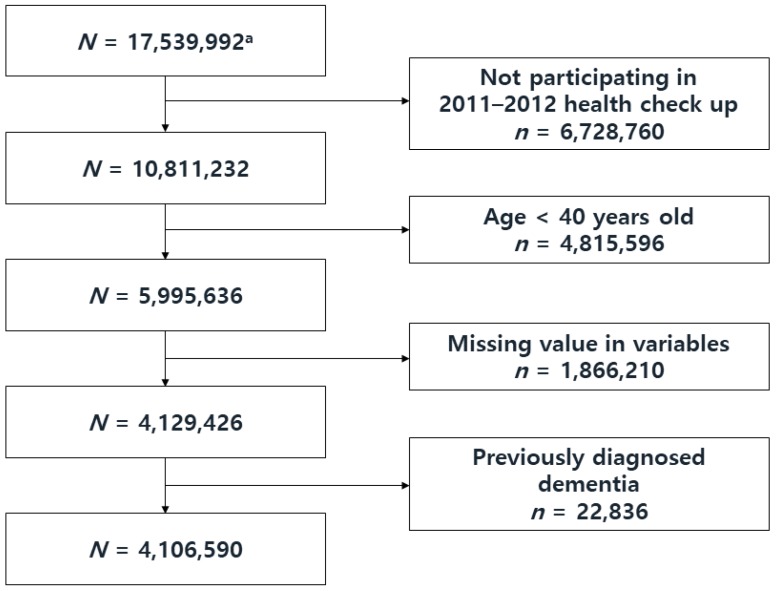
Flow Chart of the Study Population Selection Process. ^a^ Initial population: Participants of the National Health Screening Program in 2009–2010.

The initial population was NHSP participants (Korean population aged 40 and above, and all employees regardless of age) in 2009–2010 (*n* = 17,539,992). Those who did not participate in subsequent 2011–2012 NHSP (*n* = 6,728,760), and those younger than 40 years old at baseline (*n* = 4,815,596) were excluded. Participants with a history of all-cause dementia (International Classification of Disease, 10th Revision (ICD-10) codes: F00, F01, F02, F03, G23.1, G30, G31) before the index date, which was the second NHSP examination day in 2011–2012 (*n* = 22,836) were excluded. Participants with any missing variable values (e.g., past medical history or lifestyle questionnaire, *n* = 1,866,210) were also excluded. Finally, 4,106,590 participants were included in analyses (Figure 1). This study was approved by the Institutional Review Board of Samsung Medical Center (IRB File No. SMC 2019-01-026).

### 2.3. Variables 

#### 2.3.1. Independent Variables 

Information pertaining to MS was obtained from anthropometric measurements and laboratory tests from NHSP, measured according to the screening protocol. Waist circumference was measured at the midpoint between the bottom of the last rib and the top of the iliac crest at the midaxillary line. Brachial blood pressure was measured by a trained clinician after participants were seated for 5 min with an arm in the appropriate position. Blood glucose, triglyceride, and HDL levels were from blood samples after overnight fast. The definition of MS followed the 2009 agreement of the International Diabetes Federation and American Heart Association/National Heart, Lung, and Blood Institute [5]. By definition, the presence of three or more out of five risk factors constituted an MS diagnosis: triglycerides ≥ 150 mg/dL, HDL < 40 mg/dL in men and < 50 mg/dL in women, systolic blood pressure ≥ 130 mmHg and/or diastolic blood pressure ≥ 85 mmHg, fasting glucose ≥ 100 mg/dL, and abdominal obesity. Abdominal obesity was defined as waist circumference ≥ 90 cm for men and ≥ 85 cm for women, according to the definition from the Korean Society for the Study of Obesity [16].

We compared 2009–2010 and 2011–2012 NHSP results. Using MS change during biennial screening, we divided participants into four groups: sustained normal, worsened, improved, and sustained MS. 

#### 2.3.2. Outcome Variable

The outcome of this study was newly diagnosed dementia, defined as antidementia drugs prescribed at least twice with codes for AD (ICD-10 F00 or G30), VD (ICD-10 F01), or other dementia (ICD-10 F02, F03, G23.1 or G31). Antidementia drugs included acetylcholinesterase inhibitors (donepezil hydrochloride, rivastigmine, galantamine) and an NMDA receptor antagonist (memantine). In Korea, to file expense claims for drug prescriptions, physicians must document evidence of cognitive dysfunction according to the National Health Insurance Reimbursement criteria: MMSE ≤ 26 and either Clinical Dementia Rating ≥ 1 or Global Deterioration Scale ≥ 3. 

### 2.4. Covariates

Previously known dementia risk factors—smoking, alcohol consumption, exercise, stroke, depression, and chronic kidney disease (CKD)—were included in analyses. Information for smoking, alcohol, and exercise was from questionnaires administered on the index date. Smoking was divided into nonsmoking, past, and current. Alcohol consumption was divided into three levels: nondrinking, mild-to-moderate drinking as average <30 g/day, and heavy drinking as ≥30 g/day. Regular exercise was defined as ≥30 min of moderate physical activity ≥five times per week or ≥20 min of strenuous physical activity ≥three times per week. Stroke was defined by self-reported past medical history at index date. Depression was defined if ICD-code F32 or F33 was diagnosed before index date. CKD was defined as an estimated glomerular filtration rate <60 mL/min/1.73 m^2^ by the modification of diet in renal disease equation from a blood test.

### 2.5. Statistical Analyses

By the MS change groups (sustained normal, worsened, improved, and sustained MS) clinical characteristics were summarized as numbers with percentages for categorical variables and mean values with standard deviations for continuous variables. For each characteristic, we performed groupwise comparisons using chi-squared tests for categorical variables and two-tailed Student’s *t*-tests for continuous variables (Table 1). For continuous variables, statisticians confirmed the normal distribution using pictures such as the Q-Q PLOT. Statistical differences between groups were represented by *p*-Values.

Survival analysis was performed to determine the longitudinal relationship between MS and dementia incidence. Patients were followed from index date to occurrence of dementia, death, or last follow-up day (31 December 2016), whichever came first. We compared the incidence of dementia by MS status at index date, then incidence of dementia for the four MS change groups. Multivariate analysis included smoking, alcohol consumption, regular exercise, stroke, depression, and CKD. Because in previous studies, MS had different effects on dementia occurrence according to age groups [4,8], stratified analysis was done by dividing participants into age groups of <65 and ≥65 years. The Cox proportional hazards model was used to estimate hazard ratios (HR) and 95% confidence intervals (95% CIs). SAS version 9.4 (SAS Institute Inc., Cary, NC, USA) was used for all statistical analyses and *p*-Value < 0.05 was considered to indicate statistical significance.

## 3. Results

### 3.1. Study Population Characteristics

Among the 4,106,590 total participants, 2,226,415 remained normal during the 2009–2010 and 2011–2012 NHSP (sustained normal group). Newly developed MS was seen in 489,159 in the second screening (worsened group) and 381,311 had MS at the first screening that normalized at the second screening (improved group). Sustained MS was noted for 1,009,705 during the two screenings (sustained MS group). Heavy drinking was higher in the worsened (6.7%), improved (6.5%), and sustained MS group (6.2%) than the sustained normal group (5.0%). The regular exercise rate was higher in the sustained normal group (58.1%) than in other groups. Stroke (3.0%), depression (7.4%), and CKD (10.7%) were higher in the sustained MS group. All characteristics were significantly different among the four groups (*p* < 0.001) (Table 1).

### 3.2. Risk of Dementia According to Baseline Metabolic Syndrome and Components

Mean follow-up was 4.9 years. For total dementia, the incident rate for the normal group was 2.92 cases per 1,000 person-years and 6.80 cases in the MS group. Dementia occurrence risk was higher in the MS group after adjustment for age, sex, smoking, alcohol, regular exercise, stroke, depression, and CKD (Model 2) (adjusted hazard ratio (aHR), 1.12; 95% CI, 1.11–1.14). All MS components also showed higher risk of total dementia occurrence. 

AD occurrence risk was higher in the MS group (1.09; 1.07–1.11). All components of MS except waist circumference were associated with a higher risk of AD occurrence. VD occurrence risk was higher in the MS group (1.27; 1.22–1.32) and all MS components were associated with a higher risk of VD occurrence (Table 2).

### 3.3. Risk of Dementia According to Change in Baseline Metabolic Syndrome and Components

Compared to the sustained normal group, other groups showed a higher risk of total dementia, AD, and VD. Adjusted hazard ratios (aHRs) and 95% CIs for total dementia, AD, and VD were 1.11 (1.08–1.13), 1.08 (1.05–1.11), and 1.20 (1.13–1.28) in the worsened group; 1.12 (1.10–1.15), 1.10 (1.07–1.13), and 1.19 (1.12–1.27) in the improved group; and 1.18 (1.16–1.20), 1.13 (1.11–1.15), and 1.38 (1.32–1.44) in the sustained MS group.

**Figure 2:**
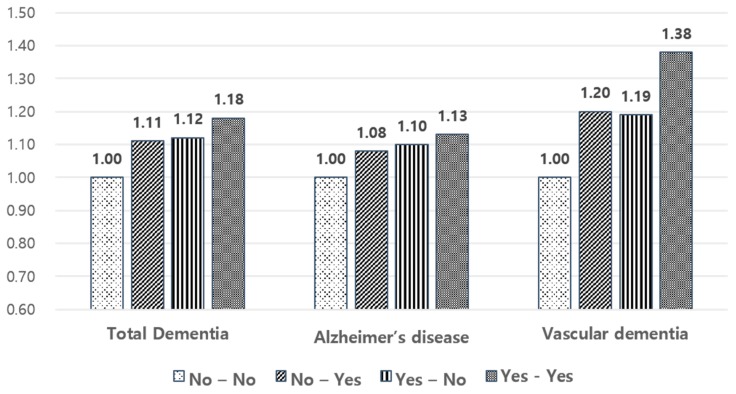
Risk of dementia occurrence according to change in metabolic syndrome status.

Normalization of MS, comparing sustained MS to the improved group, had a lowered risk of total dementia (aHR 1.18 versus 1.12), AD (1.13 versus 1.10), and VD (1.38 versus 1.19). Normalization of blood pressure (1.16 versus 1.13) and fasting glucose (1.27 versus 1.05) especially reduced the risk of dementia (Table 3, Figure 2).

### 3.4. Risk of Dementia According to Change in Baseline Metabolic Syndrome and Components in Participants <65 and ≥65 Years

Stratified analyses by age showed that change in MS status and its components had a larger association with dementia occurrence in younger than older participants. For example, in participants <65 years old compared to the sustained normal group, the risk of total dementia was higher in the worsened (aHR 1.16; 95% CI 1.10–1.23), improved (1.18; 1.11–1.25), and sustained MS (1.28; 1.23–1.34) groups. Risks were higher than those for participants ≥65 years old.

Normalization of MS also lowered risk of total dementia (aHR 1.28 versus 1.18), AD (1.21 versus 1.14), and VD (1.57 versus 1.27) in participants <65 years old. Also, in participants ≥65 years old, normalization of MS lowered total dementia (1.08 versus 1.06) and VD (1.23 versus 1.11) (Table 4).

## 4. Discussion

To the best of our knowledge, this is the first study to show the association between changes in MS and each component status and risk of dementia occurrence, which was divided into dementia subtypes. We also showed that the improvement of MS was associated with a reduction in subsequent dementia. We demonstrated that, among the MS components, fasting glucose and blood pressure were most strongly associated with risk of dementia.

In this study, MS increased the risk of both AD and VD. MS was more strongly associated with VD (aHR 1.27, 95% CI 1.22–1.32) than AD (aHR 1.09, 95% CI, 1.07–1.11). This result was consistent with previous studies [9,17], in which VD was more strongly associated with MS than AD. The causes of VD include small strokes, large infarctions, and small vessel disease, all of which are affected by cardiovascular risk factors. The importance of vascular components in the development of cognitive decline and dementia has been widely recognized, so cognitive disorders associated with cerebrovascular disease are called vascular cognitive impairment (VCI), with VD the most severe form [18]. This study result supports the recent concept of VCI.

Our study also showed that MS increased the risk of not only VD but also AD. In some previous studies, AD occurrence was also affected by MS [19,20]. This association is explained by several mechanisms. In hyperglycemia, insulin resistance stimulates amyloid β deposition, which is a key mechanism of AD [21]. Small vessel damage by hyperglycemia is thought to disturb blood supply in the brain, and glycation induces neurodegeneration, which results in brain atrophy and AD [22]. Furthermore, with high blood pressure, overactivation of the renin–angiotensin system is considered to contribute to AD pathogenesis. Our study also showed that hyperglycemia, and to a lesser degree, high blood pressure, high triglycerideTG, and low HDL, were associated with increased AD risk.

However, whether improvement in MS actually lowers dementia risk has been unclear. To date, only a few studies have shown a relationship between change in MS status and dementia. We showed that an improvement of MS status was associated with reduced occurrence of dementia. This result is consistent with a previous study [13], in which an improved group showed less risk of dementia than a persistent MS group. Furthermore, we demonstrated that among MS components, fasting glucose and blood pressure were most important for risk reduction. While causality could not be confirmed by this observational study, the result implied that improvement of MS and its components is a good strategy to prevent dementia. In a recent large trial, the FINGER study [23], a multidomain lifestyle intervention program including nutrition, exercise, and cognitive training focusing on metabolic risk management improved the cognitive function of at-risk older people. Also, cardiovascular medication, especially antihypertensive medication, showed some benefit for dementia risk [24]. Diabetes mellitus and hyperlipidemia have less evidence, however. Antidiabetic medication and statins also show the possibility of reducing dementia risk in some studies [25,26]. Thus, our study is consistent with previous studies and suggests the improvement of MS as a target to prevent dementia.

In stratified analyses, we investigated the relative magnitude of the association of MS status and its change and dementia in younger and older age groups. The impact of MS and each MS component was generally larger in the younger than the older population, especially for VD (aHR 1.57 versus 1.23). In many previous studies, midlife cardiovascular risk factors were associated with risk of dementia in late life [27,28]. Also, our study implied that MS may be a risk factor for early onset dementia at a younger age and intensive management of MS at midlife could be an effective strategy to prevent it. Therefore, these results show that control of metabolic risk factors, especially at a young age, is important for preventing dementia.

Meanwhile, the lesser impact of MS components in older age groups could be interpreted, in part, by the reverse epidemiology of cardiovascular risk factors. Some degrees of elevated body mass index, serum cholesterols, and blood pressure are reportedly associated with lower, instead of higher, risk of death among the elderly. This phenomenon is termed reverse epidemiology. [29] This relationship may be applied to dementia, explaining the current results, because dementia is also influenced by cardiovascular risk factors.

In older populations, only blood glucose is consistently associated with cognitive decline. Other cardiovascular risk factors have shown more mixed results [30]. The reason for this is thought to be because in older populations, normal aging, co-morbidities, and other changes are connected to cognitive decline, making associations more complex. In our study, even though the effects were smaller than in the younger group, improvement in fasting glucose and blood pressure reduced dementia risk in the older group. Thus, we suggest that improvement of cardiovascular risk factors is important for prevention of dementia, even in late life.

In studying MS components, improvement of blood glucose mostly lowered the risk of dementia (aHR 1.27 in persistent MS versus 1.05 in improved group), including both AD (1.26 versus 1.04) and VD (1.35 versus 1.04). In previous studies, hyperglycemia was most consistently associated with cognitive decline compared to other MS components [11,31]. As an underlying mechanism, in insulin resistance conditions, which are closely related with hyperglycemia, increased content of oxidative modification products have been reported in serum samples. Of all the body organs, the brain, including the cortex and hippocampus, is particularly sensitive to free radical attack. [32] This is thought to increase the risk of cerebral degeneration, cognitive impairment, and Alzheimer’s disease, explaining the relationship between hyperglycemia and risk of dementia. However, few studies have investigated the effect of blood glucose change on dementia incidence. Our study suggested that an improvement in blood glucose could be an important strategy for dementia prevention.

In addition, the improvement of blood pressure also lowered risk of VD (aHR 1.70 for persistent MS versus 1.39 for improved group), but this effect was not distinct for AD (1.09 versus 1.08). This finding was consistent with a previous result that blood pressure is more strongly correlated with VD than AD risk [33]. This result could be explained because stroke is a cause of VD and hypertension is a strong stroke risk factor. In several studies, antihypertensive medication use has been associated with less cognitive decline [24,34]. Our study reaffirmed that lowering blood pressure helps prevent dementia, especially VD.

Our study showed that improvement in triglyceride (aHR 1.11 for persistent versus 1.12 for improvement group) and HDL (1.10 versus 1.10) was not associated with decreased dementia risk. Results are mixed for dyslipidemia, use of lipid–lowering agents, and the risk of dementia [35,36]. These complex relationships might be the reason for the null association between improvement in lipid profile and dementia risk.

Unlike other components, waist circumference showed heterogeneous results by age group. In younger patients, a decrease in waist circumference was associated with a decreased risk of VD (aHR 1.25 for persistently increased versus 1.17 for waist circumference reduction group). This association was not found for AD. In contrast, in older people, an increase in waist circumference was associated with lower AD risk (aHR 0.96, 95% CI 0.93–0.99), and decrease in waist circumference was associated with higher AD risk (0.94 versus 1.02) and VD (1.07 versus 1.12). These contradictory findings are supported by previous studies: some showed that midlife central obesity increases risk of dementia [28,37]. However, other studies on late-life waist circumferences showed neutral [38] or opposite results [17]. This contradiction is because patients might lose weight before AD diagnosis, so waist circumference change in older adults with dementia could be interpreted as a prodrome of the disease rather than cause.

This study had some limitations. First, we examined change in MS at health screenings at only a two-year interval, so we could not include long-term changes in MS. Longer sustained changes could be associated with even higher or lower risk of dementia. Second, our study could not include baseline cognitive function and some risk factors of dementia, such as educational status and genetic factors. Third, follow-up was relatively short, considering the time for dementia occurrence. However, the higher risk of dementia could be explained by a change in MS status that affected the progression or worsening of dementia.

## 5. Conclusions

In conclusion, this study reaffirmed that MS increased the risk of dementia and showed that MS improvement reduced subsequent dementia occurrence. Among MS components, improvements in hyperglycemia and blood pressure were most effective. Therefore, it is necessary to emphasize the improvement of MS, especially hyperglycemia and blood pressure, to prevent dementia. Strategies to improve MS may help prevent dementia occurrence.

## Figures and Tables

**Table 1:** Study population characteristics according to baseline and change in metabolic syndrome.

	Total	Normal	Normal	MS	MS *	*p*-Value ^†^
		Normal	MS	Normal	MS	
*N*	4,106,590	2,226,415	489,159	381,311	1,009,705	
Age, mean ± SD	55.8 ± 10.1	53.2 ± 9.3	56.9 ± 10.0	57.3 ± 10.2	60.3 ± 10.1	<0.001
Sex, *n* (%)						
Male	2,237,407(54.5)	1,223,715(55.0)	278,150(56.9)	222,657(58.4)	512,885(50.8)	<0.001
Female	1,869,183(45.5)	1,002,700(45.0)	211,009(43.1)	158,654(41.6)	496,820(49.2)	
Smoking ^‡^, *n* (%)						
Never	2,504,680(61.0)	1,362,613(61.2)	286,876(58.7) **	221,664(58.1)	633,527(62.7)	<0.001
Former	806,746(19.6)	423,281(19.0)	103,070(21.1)	80,408(21.1)	199,987(19.8)	
Current	795,164(19.4)	440,521(19.8)	99,213(20.3) ^††^	79,239(20.8)	176,191(17.5)	
Alcohol ^§^, *n* (%)						
None	2,312,346(56.3)	1,206,166(54.2)	270,999(55.4)	211,704(55.5)	623,477(61.8)	<0.001
Mild-to-moderate	1,563,365(38.1)	909,381(40.9)	185,331(37.9)	144,676(37.9)	323,977(32.1)	
Heavy	230,879(5.6)	110,868(5.0) ^††^	32,829(6.7)	24,931(6.5) ^††^	62,251(6.2) ^††^	
Regular exercise ^||^, *n* (%)						
No	1,833,179(44.6)	932,926(41.9)	226,763(46.4)	172,660(45.3)	500,830(49.6)	<0.001
Yes	2,273,411(55.4)	1,293,489(58.1)	262,396(53.6)	208,651(54.7)	508,875(50.4)	
Stroke ^¶^, *n* (%)						
No	4,045,368(98.5)	2,210,033(99.3)	480,450(98.2)	375,107(98.4)	979,778(97.0)	<0.001
Yes	61,222(1.5)	16,382(0.7)	8709(1.8)	6204(1.6)	29,927(3.0)	
Depression ^#^, *n* (%)						
No	3,900,412(95.0)	2,143,113(96.3)	461,028(94.2)	360,984(94.7)	935,287(92.6)	<0.001
Yes	206,178(5.0)	83,302(3.7)	28,131(5.8)	20,327(5.3)	74,418(7.4)	
CKD **, *n* (%)						
No	3,863,020(94.1)	2,144,661(96.3)	458,555(93.7)	357,864(93.9)	901,940(89.3)	<0.001
Yes	243,570(5.9)	81,754(3.7)	30,604(6.3)	23,447(6.2) ^††^	107,765(10.7)	

Abbreviations: MS = metabolic syndrome, CKD = chronic kidney disease. * MS was defined from blood tests and anthropometric measurements from 2009–2010 and 2011–2012 national health examinations. ^†^ Comparison was by Student’s *t*-test for continuous variables and chi-squared test for categorical variables. ^‡^ Smoking history was divided into nonsmokers, ex-smokers, and current smokers based on questionnaires from the 2011–2012 examination. ^§^ Mild-to-moderate drinking was defined as less than 30 g of alcohol per day, and heavy drinking as 30 g or more, based on questionnaires from the 2011–2012 examination. ^||^ Regular exercise was defined as ≥30 min of moderate physical activity ≥five times per week or ≥20 min of strenuous physical activity ≥three times per week.^¶^ Stroke was defined from past medical history screening from the 2011–2012 examination. ^#^ Depression was defined as ICD-code F32 or F33 diagnosed in 2011–2012, before index date. ** CKD was defined as estimated glomerular filtration rate <60 mL/min/1.73 m^2^ by modification of diet in renal disease equation from blood tests from the 2011–2012 examination. ^††^ Total percentages may not equal 100% because of rounding.

**Table 2:** Occurrence of dementia according to baseline metabolic syndrome and components.

	Total Dementia *			Alzheimer’s Disease *			Vascular Dementia *		
2009–2010	Rate	Model 1 ^‡^	Model 2 ^§^	Rate	Model 1 ^‡^	Model 2 ^§^	Rate	Model 1 ^‡^	Model 2 ^§^
To 2011–2012	(1/1000)	aHR	aHR	(1/1000)	aHR	aHR	(1/1000)	aHR	aHR
		(95% CI)	(95% CI)		(95% CI)	(95% CI)		(95% CI)	(95% CI)
MS ^†^									
No	2.92	1.00	1.00	2.21	1.00	1.00	0.36	1.00	1.00
Yes	6.80	1.81	1.12	5.10	1.14	1.09	0.89	1.37	1.27
		(1.17–1.20)	(1.11–1.14)		(1.12–1.16)	(1.07–1.11)		(1.32–1.43)	(1.22–1.32)
WC ^†^									
No	3.42	1.00	1.00	2.57	1.00	1.00	0.44	1.00	1.00
Yes	6.32	1.03	1.01	4.77	1.00	0.99	0.80	1.14	1.11
		(1.02–1.04)	(1.00–1.03)		(0.99–1.02)	(0.97–1.00)		(1.09–1.18)	(1.07–1.15)
Blood pressure ^†^									
No	2.16	1.00		1.66	1.00	1.00	0.24	1.00	1.00
Yes	6.20	1.13	1.11	4.64	1.07	1.06	0.82	1.52	1.47
		(1.11–1.15)	(1.09–1.13)		(1.05–1.09)	(1.04–1.08)		(1.45–1.59)	(1.40–1.54)
Fasting glucose ^†^									
No	3.44	1.00	1.00	2.61	1.00	1.00	0.43	1.00	1.00
Yes	5.76	1.22	1.20	4.31	1.20	1.19	0.75	1.29	1.26
		(1.20–1.24)	(1.19–1.22)		(1.19–1.22)	(1.17–1.21)		(1.24–1.34)	(1.22–1.31)
Triglyceride ^†^									
No	3.66	1.00	1.00	2.78	1.00	1.00	0.46	1.00	1.00
Yes	5.27	1.14	1.08	3.94	1.11	1.06	0.69	1.24	1.14
		(1.12–1.15)	(1.06–1.09)		(1.09–1.13)	(1.04–1.08)		(1.19–1.28)	(1.10–1.18)
HDL ^†^									
No	3.22	1.00	1.00	2.42	1.00	1.00	0.42	1.00	1.00
Yes	6.41	1.15	1.08	4.85	1.13	1.07	0.81	1.24	1.13
		(1.13–1.16)	(1.06–1.09)		(1.11–1.15)	(1.05–1.09)		(1.19–1.29)	(1.09–1.18)

Abbreviations: MS = metabolic syndrome, WC = waist circumference, HDL = high density lipoprotein, aHR = adjusted hazard ratio, CI = confidence interval. Participants were followed from the 2009–2010 and the 2011–2012 national examination to 31 December 2016. * Occurrence of dementia was defined as antidementia drug prescription at least twice with codes for Alzheimer’s disease (ICD-10 F00 or G30), vascular dementia (ICD-10 F01), or other dementia (ICD-10 F02, F03, G23.1 or G31). ^†^ MS and components were defined from blood tests and anthropometric measurements from 2009–2010 and 2011–2012 examinations: waist circumference ≥ 90 cm for men and ≥ 85 cm for women, systolic blood pressure ≥ 130 mmHg and/or diastolic blood pressure ≥ 85 mmHg, fasting glucose ≥ 100 mg/dL, triglycerides ≥ 150 mg/dL, HDL < 40 mg/dL in men and < 50 mg/dL in women. The presence of three or more out of five factors was regarded as MS. ^‡^ Model 1 includes age and sex. ^§^ Model 2 includes age, sex, smoking, alcohol, regular exercise, stroke, depression, and chronic kidney disease.

**Table 3:** Occurrence of dementia according to change in metabolic syndrome and components.

	Total Dementia *			Alzheimer’s Disease *			Vascular Dementia *		
2009–2010	Rate	Model 1 ^‡^	Model 2 ^§^	Rate	Model 1 ^‡^	Model 2 ^§^	Rate	Model 1 ^‡^	Model 2 ^§^
to 2011–2012	(1/1000)	aHR	aHR	(1/1000)	aHR	aHR	(1/1000)	aHR	aHR
		(95% CI)	(95% CI)		(95% CI)	(95% CI)		(95% CI)	(95% CI)
MS ^†^									
No–No	2.52	1.00	1.00	1.91	1.00	1.00	0.32	1.00	1.00
No–Yes	4.86	1.15	1.11	3.65	1.12	1.08	0.62	1.27	1.20
		(1.13–1.18)	(1.08–1.13)		(1.09–1.15)	(1.05–1.11)		(1.20–1.35)	(1.13–1.28)
Yes–No	5.26	1.15	1.12	3.98	1.13	1.10	0.65	1.24	1.19
		(1.13–1.18)	(1.10–1.15)		(1.10–1.16)	(1.07–1.13)		(1.16–1.33)	(1.12–1.27)
Yes–Yes	7.75	1.25	1.18	5.80	1.20	1.13	1.02	1.51	1.38
		(1.23–1.27)	(1.16–1.20)		(1.18–1.22)	(1.11–1.15)		(1.45–1.58)	(1.32–1.44)
WC ^†^									
No–No	3.09	1.00	1.00	2.32	1.00	1.00	0.40	1.00	1.00
No–Yes	4.72	1.03	1.02	3.52	1.00	0.99	0.60	1.12	1.09
		(1.01–1.06)	(0.99–1.04)		(0.97–1.03)	(0.96–1.02)		(1.04–1.19)	(1.02–1.17)
Yes–No	5.77	1.09	1.07	4.37	1.07	1.06	0.73	1.19	1.17
		(1.07–1.12)	(1.05–1.10)		(1.05–1.10)	(1.03–1.08)		(1.12–1.27)	(1.09–1.24)
Yes–Yes	6.96	1.06	1.03	5.27	1.03	1.00	0.88	1.20	1.16
		(1.04–1.07)	(1.02–1.05)		(1.01–1.05)	(0.99–1.02)		(1.15–1.26)	(1.11–1.22)
Blood pressure ^†^									
No–No	1.73	1.00	1.00	1.34	1.00	1.00	0.19	1.00	1.00
No–Yes	3.75	1.16	1.15	2.84	1.11	1.09	0.47	1.51	1.48
		(1.13–1.19)	(1.12–1.18)		(1.07–1.14)	(1.06–1.13)		(1.40–1.64)	(1.37–1.61)
Yes–No	3.66	1.14	1.13	2.78	1.09	1.08	0.44	1.41	1.39
		(1.10–1.17)	(1.09–1.16)		(1.05–1.13)	(1.05–1.12)		(1.30–1.54)	(1.28–1.51)
Yes–Yes	6.92	1.19	1.16	5.17	1.11	1.09	0.93	1.78	1.70
		(1.17–1.22)	(1.14–1.19)		(1.08–1.13)	(1.06–1.11)		(1.67–1.89)	(1.60–1.81)
Fasting glucose ^†^									
No–No	3.28	1.00	1.00	2.48	1.00	1.00	0.41	1.00	1.00
No–Yes	4.41	1.10	1.08	3.31	1.08	1.07	0.56	1.12	1.11
		(1.07–1.12)	(1.06–1.11)		(1.06–1.11)	(1.05–1.10)		(1.06–1.19)	(1.04–1.17)
Yes–No	4.14	1.05	1.05	3.13	1.04	1.04	0.51	1.04	1.04
		(1.02–1.07)	(1.02–1.07)		(1.01–1.07)	(1.01–1.07)		(0.97–1.11)	(0.98–1.11)
Yes–Yes	6.49	1.29	1.27	4.86	1.27	1.26	0.86	1.38	1.35
		(1.27–1.31)	(1.25–1.29)		(1.25–1.30)	(1.23–1.28)		(1.32–1.44)	(1.29–1.41)
Triglyceride ^†^									
No–No	3.36	1.00	1.00	2.55	1.00	1.00	0.41	1.00	1.00
No–Yes	4.76	1.17	1.12	3.59	1.15	1.11	0.59	1.21	1.13
		(1.14–1.19)	(1.10–1.14)		(1.12–1.18)	(1.08–1.13)		(1.14–1.28)	(1.07–1.20)
Yes–No	4.94	1.14	1.11	3.72	1.12	1.09	0.64	1.24	1.18
		(1.12–1.17)	(1.08–1.13)		(1.10–1.15)	(1.06–1.12)		(1.16–1.31)	(1.11–1.25)
Yes–Yes	5.50	1.18	1.10	4.10	1.14	1.07	0.74	1.34	1.21
		(1.16–1.20)	(1.08–1.12)		(1.12–1.16)	(1.05–1.09)		(1.28–1.40)	(1.16–1.26)
HDL ^†^									
No–No	2.84	1.00	1.00	2.13	1.00	1.00	0.37	1.00	1.00
No–Yes	5.28	1.18	1.12	3.97	1.16	1.10	0.69	1.28	1.19
		(1.16–1.20)	(1.09–1.14)		(1.13–1.19)	(1.08–1.13)		(1.21–1.36)	(1.12–1.26)
Yes–No	5.17	1.17	1.12	3.90	1.15	1.10	0.65	1.24	1.17
		(1.15–1.20)	(1.10–1.15)		(1.12–1.18)	(1.08–1.13)		(1.16–1.32)	(1.10–1.24)
Yes–Yes	7.06	1.20	1.11	5.34	1.18	1.10	0.89	1.32	1.17
		(1.18–1.22)	(1.09–1.13)		(1.16–1.20)	(1.08–1.12)		(1.26–1.38)	(1.12–1.23)

Abbreviations: MS = metabolic syndrome, WC = waist circumference, HDL = high density lipoprotein, aHR = adjusted hazard ratio, CI = confidence interval. Participants were followed from the 2009–2010 and 2011–2012 national examination to 31 December 2016. * Occurrence of dementia was defined as antidementia drugs prescribed at least twice with codes for Alzheimer’s disease (ICD–10 F00 or G30), vascular dementia (ICD–10 F01), or other dementia (ICD–10 F02, F03, G23.1 or G31). ^†^ MS and components were defined from blood tests and anthropometric measurements of 2009–2010 and 2011–2012 examination: waist circumference ≥ 90 cm for men and ≥ 85 cm for women, systolic blood pressure ≥ 130 mmHg and/or diastolic blood pressure ≥ 85 mmHg, fasting glucose ≥ 100 mg/dL, triglycerides ≥ 150 mg/dL, HDL < 40 mg/dL in men and < 50 mg/dL in women. The presence of three or more out of five factors was regarded as MS. ^‡^ Model 1 includes age and sex. ^§^ Model 2 includes age, sex, smoking, alcohol, regular exercise, stroke, and chronic kidney disease.

**Table 4:** Stratified analysis by participant age (<65 versus ≥65 years).

	Total Dementia *			Alzheimer’s Disease *		Vascular Dementia *	
	All	<65	≥65	<65	≥65	<65	≥65
	aHR	aHR	aHR	aHR	aHR	aHR	aHR ^‡^
	(95% CI)	(95% CI)	(95% CI)	(95% CI)	(95% CI)	(95% CI)	(95% CI)
MS ^†^							
No–No	1.00	1.00	1.00	1.00	1.00	1.00	1.00
No–Yes	1.11	1.16	1.05	1.12	1.02	1.27	1.12
	(1.08–1.13)	(1.10–1.23)	(1.02–1.07)	(1.05–1.20)	(1.00–1.05)	(1.12–1.43)	(1.04–1.20)
Yes–No	1.12	1.18	1.06	1.14	1.05	1.27	1.11
	(1.10–1.15)	(1.11–1.25)	(1.04–1.09)	(1.06–1.23)	(1.02–1.08)	(1.11–1.46)	(1.03–1.19)
Yes–Yes	1.18	1.28	1.08	1.21	1.05	1.57	1.23
	(1.16–1.20)	(1.23–1.34)	(1.06–1.10)	(1.15–1.27)	(1.03–1.07)	(1.43–1.72)	(1.17–1.30)
WC ^†^							
No–No	1.00	1.00	1.00	1.00	1.00	1.00	1.00
No–Yes	1.02	1.01	0.99	0.98	0.96	1.12	1.05
	(0.99–1.04)	(0.95–1.08)	(0.96–1.01)	(0.91–1.05)	(0.93–0.99)	(0.97–1.28)	(0.97–1.13)
Yes–No	1.07	1.11	1.03	1.09	1.02	1.17	1.12
	(1.05–1.10)	(1.04–1.17)	(1.01–1.06)	(1.02–1.18)	(0.99–1.05)	(1.02–1.34)	(1.05–1.21)
Yes–Yes	1.03	1.08	0.97	1.03	0.94	1.25	1.07
	(1.02–1.05)	(1.03–1.12)	(0.95–0.98)	(0.98–1.08)	(0.92–0.96)	(1.14–1.38)	(1.02–1.13)
Blood pressure ^†^							
No–No	1.00	1.00	1.00	1.00	1.00	1.00	1.00
No–Yes	1.15	1.17	1.07	1.07	1.04	1.68	1.28
	(1.12–1.18)	(1.10–1.25)	(1.04–1.10)	(0.99–1.15)	(1.00–1.07)	(1.46–1.94)	(1.17–1.41)
Yes–No	1.13	1.15	1.05	1.08	1.02	1.41	1.25
	(1.09–1.16)	(1.08–1.23)	(1.02–1.08)	(0.99–1.16)	(0.98–1.06)	(1.20–1.65)	(1.13–1.39)
Yes–Yes	1.16	1.26	1.06	1.10	1.00	2.03	1.42
	(1.14–1.19)	(1.21–1.31)	(1.03–1.08)	(1.05–1.16)	(0.98–1.03)	(1.83–2.26)	(1.32–1.53)
Fasting glucose ^†^							
No–No	1.00	1.00	1.00	1.00	1.00	1.00	1.00
No–Yes	1.08	1.07	1.08	1.08	1.07	1.04	1.11
	(1.06–1.11)	(1.01–1.13)	(1.06–1.11)	(1.01–1.16)	(1.04–1.09)	(0.92,1.18)	(1.04–1.19)
Yes–No	1.05	1.05	1.04	1.04	1.03	1.03	1.03
	(1.02–1.07)	(0.99–1.11)	(1.01–1.06)	(0.97–1.12)	(1.01–1.06)	(0.91,1.18)	(0.96–1.11)
Yes–Yes	1.27	1.33	1.22	1.34	1.20	1.35	1.30
	(1.25–1.29)	(1.28–1.38)	(1.20–1.24)	(1.27–1.41)	(1.18–1.23)	(1.24,1.48)	(1.23–1.36)
Triglyceride ^†^							
No–No	1.00	1.00	1.00	1.00	1.00	1.00	1.00
No–Yes	1.12	1.15	1.08	1.16	1.07	1.06	1.12
	(1.10–1.14)	(1.09–1.21)	(1.06–1.11)	(1.09–1.24)	(1.04–1.10)	(0.94–1.21)	(1.05–1.20)
Yes–No	1.11	1.15	1.07	1.12	1.06	1.28	1.12
	(1.08–1.13)	(1.09–1.22)	(1.04–1.10)	(1.04–1.20)	(1.03–1.09)	(1.13–1.45)	(1.04–1.20)
Yes–Yes	1.10	1.18	1.04	1.14	1.02	1.31	1.12
	(1.08–1.12)	(1.14–1.23)	(1.02–1.05)	(1.08–1.20)	(1.00–1.04)	(1.19–1.43)	(1.07–1.18)
HDL ^†^							
No–No	1.00	1.00	1.00	1.00	1.00	1.00	1.00
No–Yes	1.12	1.16	1.08	1.14	1.06	1.28	1.12
	(1.09–1.14)	(1.10–1.22)	(1.05–1.10)	(1.07–1.21)	(1.04–1.09)	(1.15–1.44)	(1.05–1.19)
Yes–No	1.12	1.16	1.08	1.17	1.06	1.16	1.13
	(1.10–1.15)	(1.09–1.23)	(1.06–1.11)	(1.09–1.25)	(1.04–1.09)	(1.02–1.33)	(1.05–1.21)
Yes–Yes	1.11	1.20	1.05	1.19	1.03	1.23	1.10
	(1.09–1.13)	(1.15–1.25)	(1.03–1.06)	(1.13–1.25)	(1.01–1.05)	(1.11–1.35)	(1.04–1.16)

Abbreviations: MS = metabolic syndrome, WC = waist circumference, HDL = high density lipoprotein, aHR = adjusted hazard ratio, CI = confidence interval. Participants were followed from the 2009–2010 and 2011–2012 national examination to 31 December 2016. * Occurrence of dementia was defined as antidementia drugs prescribed at least twice with codes for Alzheimer’s disease (ICD–10 F00 or G30), vascular dementia (ICD–10 F01), or other dementia (ICD–10 F02, F03, G23.1 or G31). ^†^ MS and components were defined from blood tests and anthropometric measurements of 2009–2010 and 2011–2012 examinations: waist circumference ≥ 90 cm for men and ≥ 85 cm for women, systolic blood pressure ≥ 130 mmHg and/or diastolic blood pressure ≥ 85 mmHg, fasting glucose ≥ 100 mg/dL, triglycerides ≥ 150 mg/dL, HDL < 40 mg/dL in men and < 50 mg/dL in women. The presence of three or more out of five factors was regarded as MS. ^‡^ All analyses used multivariate models including age, sex, smoking, alcohol, regular exercise, stroke, and chronic kidney disease.
